# Unilateral sublingual salivary gland hypertrophy with herniation through a boutonniére defect and contralateral sublingual gland hypoplasia

**DOI:** 10.1259/bjrcr.20150382

**Published:** 2016-07-28

**Authors:** Zenab Arooj Sher, Garryck Tan

**Affiliations:** ^1^General Surgery, Medway Maritime Hospital, Medway NHS Foundation Trust, Gillingham, UK; ^2^Radiology, Darent Valley Hospital, Dartford & Gravesham NHS Trust, Dartford, UK

## Abstract

A 74-year-old male attended our ear, nose and throat clinic with left-sided otalgia. MRI highlighted an area of abnormal signal in the region of the right mylohyoid muscle in the floor of the oral cavity. The patient was called back for a post-contrast MRI scan for clarification. Post-contrast MRI demonstrated a left-sided hypoplastic sublingual salivary gland and a hypertrophied right-sided sublingual salivary gland. The left sublingual gland hypoplasia had resulted in compensatory hypertrophy of the contralateral (right) sublingual salivary gland, extending through the mylohyoid gap or "boutonnière defect". This is a common incidental variant often encountered in radiological imaging. This is the only report of a case of contralateral compensatory hypertrophy of sublingual salivary gland, as all the others have reported ipsilateral hypertrophy of either submandibular salivary glands.

## Background

The sublingual salivary glands are located superior to the mylohyoid muscle. The sublingual glands arise from the first and second embryonic branchial arches as epithelial buds from the oral cavity. These buds grow into the surrounding mesenchymal tissue and undergo development *via* an intricate process of branching morphogenesis through epithelial to mesenchymal signalling.^1^

The mesenchyme of the mylohyoid muscle undergoes differentiation close to the developing sublingual salivary tissue. The mylohyoid muscle not only forms the floor of the mouth but also supports the tongue and defines the boundary between the submandibular and the sublingual spaces. It has anterior and posterior halves that overlap, forming a gap if the overlap is incomplete. This mylohyoid “defect” or gap is a common, incidental finding on radiological imaging. “boutonnière” means buttonhole in French, illustrating the type of defect that is encountered. It does not cause symptoms and is of little clinical significance, being present in up 77% of individuals.^2^ The mylohyoid gap is of approximately 5 mm size and can contain glandular tissue, fat or vessels.^2–4^ Failure to identify the mylohyoid gap as a normal anatomical variant on radiological imaging may provoke unnecessary investigations, anxiety and misdiagnosis, although excluding malignancy is pertinent.^5^

## Clinical presentation

A 74-year-old male non-smoker was referred to the ear, nose and throat clinic with a presenting complaint of 6 weeks of persistent sore throat and mild left-sided otalgia, with no other symptoms. Physical examination, including the mouth and oropharynx, and flexible nasendoscopy were normal.

Audiograms illustrated normal hearing thresholds within conversational range, but reduced thresholds in both ears in the higher frequencies. Tympanograms were normal.

**Figure 1.**
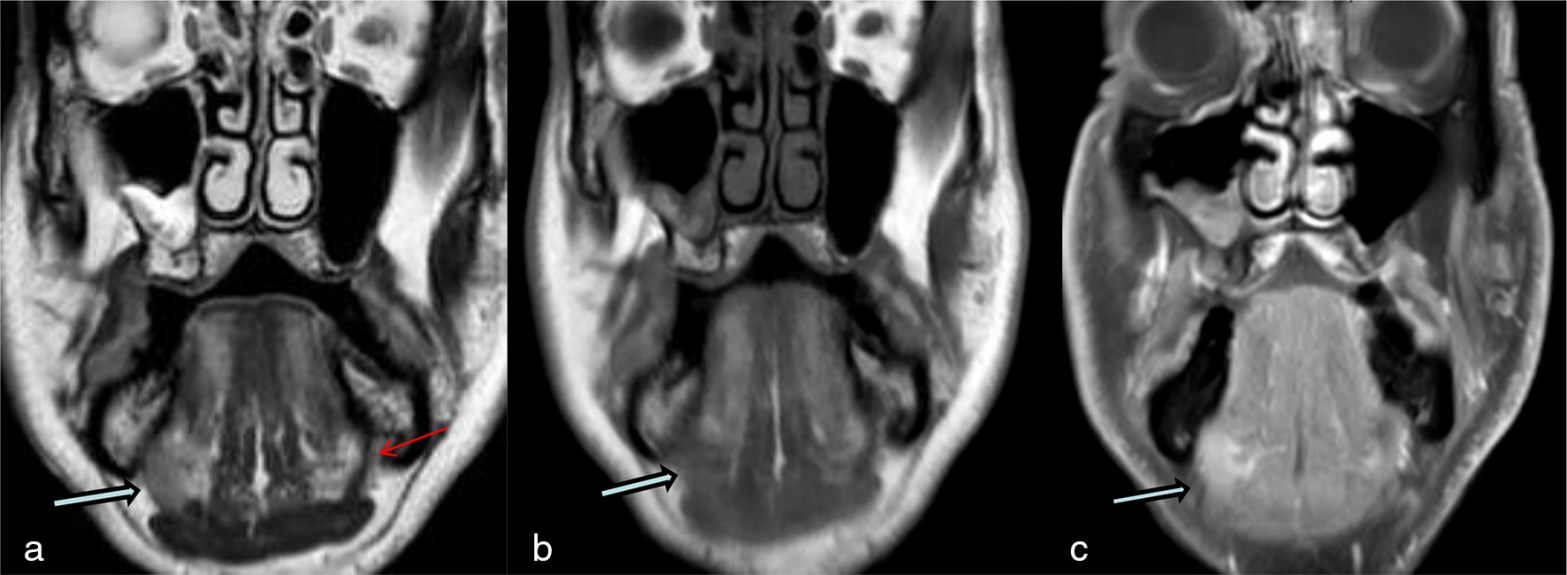
Sublingual gland hypertrophy with herniation through a mylohyoid defect. The right sublingual gland (blue arrows with black outline) within the right mylohyoid defect appears as a pseudolesion on coronal images. Note the intact left mylohyoid muscle (red arrow on a). Note also that, on *T*_1_ and *T*_2_ weighted images, the sublingual gland shows signal intensity that is higher than muscle and lower than fat. Coronal (a) *T*_2_ weighted, (b) *T*_1_ weighted and (c) *T*_1_ weighted post-gadolinium with fat saturation sequence.

Non-contrast MRI in the coronal plane showed an abnormal, high *T*_2_ signal lesion in the right mylohyoid. The axial images were, however, of suboptimal quality owing to movement artefact. The patient was recalled for post-contrast examination 2 weeks later (Figure 1).

**Figure 2.**
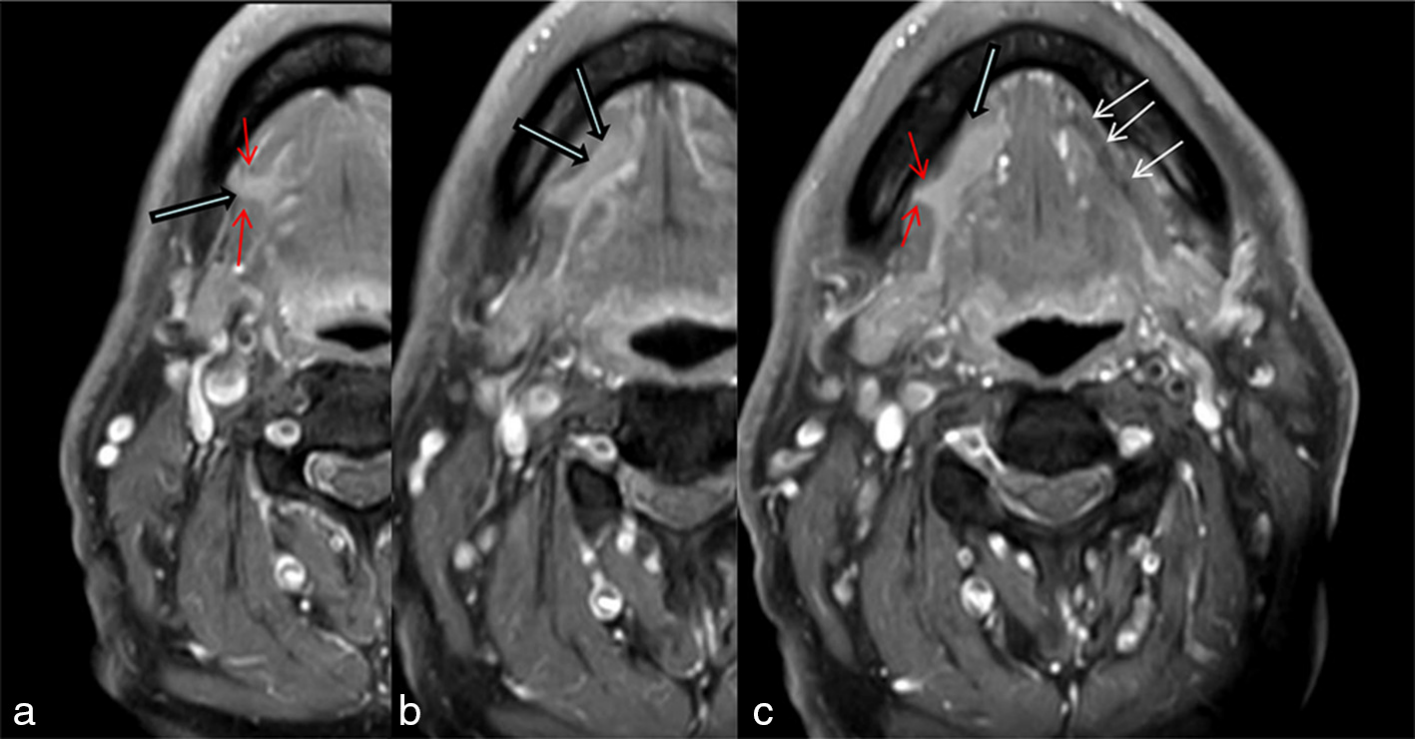
*T*_1_ weighted post-gadolinium MR contrast with fat saturation. Sequential slices (inferior to superior a, b, c) showing the right sublingual salivary gland (blue arrows with black outline) with herniation through the right mylohyoid gap (red arrows on a, c). Note the intact left mylohyoid (white arrows on c) with absent left sublingual gland.

Contrast MRI showed a hypoplastic left sublingual salivary gland, with contralateral hypertrophy of the right-sided sublingual salivary gland. The right sublingual glandular tissue was seen to be protruding through the right mylohyoid gap in the coronal section (Figure 2a) and further visualized in the axial section (Figure 2b). This “boutonnière defect” is often seen on radiological imaging and is a normal variant commonly identified on CT scan/MRI.^6–8^

**Figure 3.**
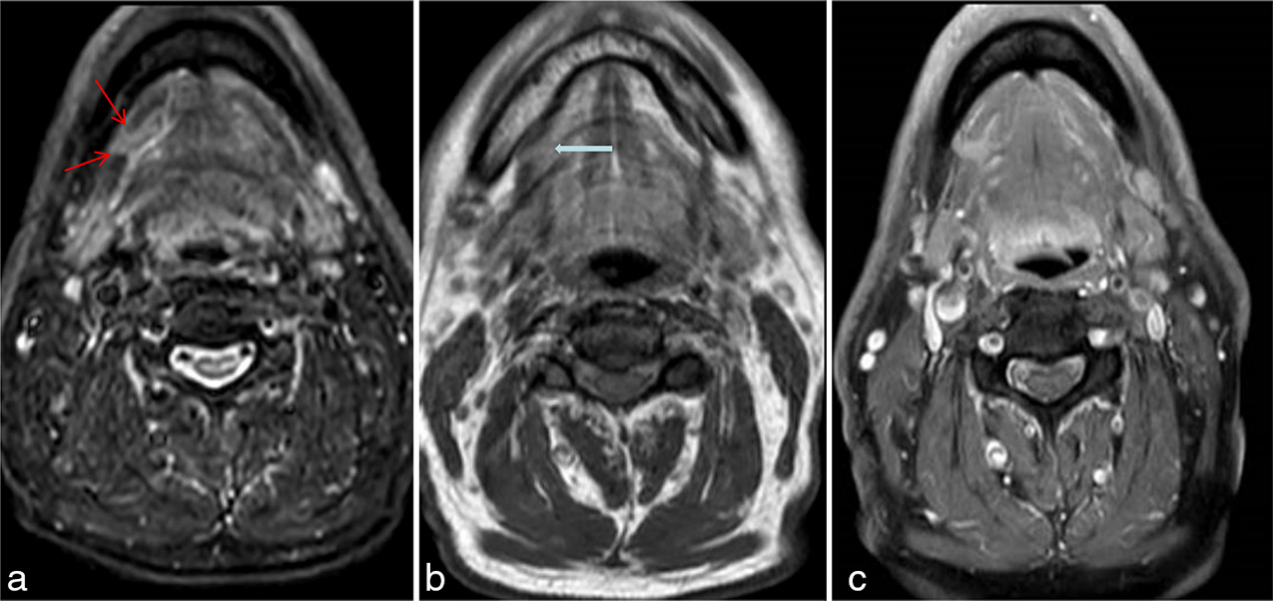
Sublingual salivary gland tissue is of higher signal intensity than muscle. The sublingual salivary gland (blue arrow with black outline on b) is of intermediate *T*_1_ signal—showing signal intensity higher than muscle and lower than fat. Mylohyoid defect is shown in (a) by red arrows. (a) Axial short *T*_1_ inversion recovery (fat saturation) sequence. (b) Axial *T*_1_ weighted sequence—there is movement artefact at the inferior tongue. (c) Axial *T*_1_ weighted post-gadolinium fat-saturated sequence.

## Differential diagnosis

Knowledge of the normal location of the sublingual gland and awareness of the mylohyoid defect should ensure that a correct diagnosis is made when sublingual gland hypertrophy is identified, with glandular tissue within a mylohyoid gap. Hypoplasia in another salivary gland will support the diagnosis. The sublingual gland shows intermediate signal intensity on *T*_1_; this signal intensity is higher than that of adjacent muscle and lower than fat (Figure 3). It should have a homogeneous unenhanced and enhanced signal, and the mylohyoid muscle should not have areas of high signal; otherwise, an alternative diagnosis such as inflammation or a neoplastic process should be considered.

## Treatment and follow-up

The patient was reassured regarding the anatomical variant in his mylohyoid muscle. His otalgia had settled, so the patient was discharged back to the care of his general practitioner with re-referral to the ear, nose and throat clinic if he became symptomatic.

## Discussion

This is the first reported case of isolated hypoplasia of a sublingual salivary gland with compensatory hypertrophy of the sublingual gland on the other side of the mouth. In all other reported cases, the hypoplastic gland is usually the submandibular salivary gland with associated ipsilateral hyperplasia of the sublingual salivary gland.^1,5,7,8^ Salivary gland agenesis is an infrequent occurrence, with only 40 cases reported to date.^1^ Agenesis may occur on a background of congenital syndromes affecting the embryonic first branchial arch, such as Treacher–Collins syndrome, or in patients who are edentulous. Complete agenesis of all salivary glands is very rare and only been reported a few times in the literature.^9^ In most cases, the patients are asymptomatic from single salivary gland agenesis, and there is usually compensatory hypertrophy of other major salivary glands.^8–10^

## Learning points

1. Knowledge of anatomy is key when reporting and analyzing images of the oral cavity.
2. It is imperative to rule out masses and lesions suspicious of malignancy.
3. However, awareness of the mylohyoid or “boutonnière defect” as a common variant through which glandular tissue may herniate is important so that incorrect diagnosis and overtreatment of incidental radiological variants is avoided.
4. Sublingual salivary gland shows intermediate signal intensity on *T*_1_ and high signal intensity on *T*_2_; the signal intensity is lower than fat and higher than muscle on both sequences (Figure 3) .

## Consent

Written informed consent was obtained from the patient for publication of this case report, including accompanying MRI images.
